# An Evaluation of the Diagnostic Accuracy of the Grade of Preoperative Biopsy Compared to Surgical Excision in Chondrosarcoma of the Long Bones

**DOI:** 10.1155/2010/270195

**Published:** 2010-04-18

**Authors:** Robert Jennings, Nicholas Riley, Barry Rose, Roberto Rossi, John A. Skinner, Steven R. Cannon, Timothy W. R. Briggs, Rob Pollock, Asif Saifuddin

**Affiliations:** ^1^The London Bone and Soft-Tissue Tumour Unit, The Royal National Orthopaedic Hospital NHS Trust, Brockley Hill, Stanmore, Middlesex HA7 4LP, UK; ^2^University of Turin Medical School, Mauriziano “Umberto I” Hospital, Largo Turati 62, 10128 Turin, Italy; ^3^Department of Diagnostic Imaging, The Royal National Orthopaedic Hospital NHS Trust, Brockley Hill, Stanmore, Middlesex HA7 4LP, UK

## Abstract

Chondrosarcoma is the second most common primary malignant bone tumour. Distinguishing between grades is not necessarily straightforward and may alter the disease management. We evaluated the correlation between histological grading of the preoperative image-guided needle biopsy and the resection specimen of 78 consecutive cases of chondrosarcoma of the femur, humerus, and tibia. In 11 instances, there was a discrepancy in histological grade between the biopsy and surgical specimen. Therefore, there was an 85.9% (67/78) accuracy rate for pre-operative histological grading of chondrosarcoma, based on needle biopsy. However, the accuracy of the diagnostic biopsy to distinguish low-grade from high-grade chondrosarcoma was 93.6% (73/78). We conclude that accurate image-guided biopsy is a very useful adjunct in determining histological grade of chondrosarcoma and the subsequent treatment plan. At present, a multidisciplinary approach, comprising experienced orthopaedic surgeons, radiologists, and pathologists, offers the most reliable means of accurately diagnosing and grading of chondrosarcoma of long bones.

## 1. Introduction

Chondrosarcoma is the second most common primary malignant bone tumour, exceeded in frequency only by osteosarcoma [1–6]. There are three well-recognised histological grades, which may influence the choice of appropriate management [7]. Unfortunately, distinguishing between grades from pre-operative image-guided biopsy is not necessarily straightforward [8–13]. Choice of management in such cases depends on a combination of history, clinical examination, and radiological findings. 

Histologically, chondrosarcomas may be classified into three groups according to cellularity and pleomorphism (I, II, and III) or may be referred to as dedifferentiated chondrosarcomas [14]. Clinically, chondrosarcomas are subdivided into low grade (I) and high grade (II, III, and dedifferentiated). Overall five-year survival has been reported as 75% [15]. Low-grade lesions tend to be slow growing, metastasise infrequently, and are associated with a 90% five-year survival rate, which plateaus after this period [16]. They are commonly treated by curettage with bone cement [17–19], although some authors describe a high mortality risk after intralesional resection (including curettage) in their reports [6, 16]. High-grade tumours have a higher incidence of metastasis, with a variably reported five-year survival rate of 40%–80%for grades II-III, but significantly inferior for dedifferentiated tumours [7, 14]. This figures plateau's at approximately 20% survival beyond 10 years [16, 20]. Most are treated with wide excision (with the margins confirmed histologically postoperatively) and reconstruction [7, 18, 21]. 

The main aim of this study was to determine the accuracy of pre-operative image-guided needle biopsy in suspected chondrosarcoma of long bones, thus allowing an evaluation of the reliability of image-guided needle biopsy for planning surgical management. We restricted the study to chondrosarcoma of the long bones given the relative ease of obtaining an adequate biopsy sample from these locations compared to those tumours located within the pelvis or other parts of the axial skeleton. Furthermore, the treatment options for such tumours may differ significantly from those located in the long bones.

## 2. Materials and Methods

We retrospectively reviewed the tumour database at a supraregional bone and soft tissue tumour unit. We examined the records of all cases of chondrosarcoma diagnosed on needle biopsy, identifying 124 patients. Cases of chondrosarcoma that did not undergo needle biopsy were excluded. Cases where the biopsy was inconclusive (the rate at our institution is 5.2% for all biopsies) or did not give a diagnosis of chondrosarcoma were not considered. We excluded tumours not located within the femur, tibia, or humerus (31 pelvic/sacral, 5 forearm/hand, 4 rib, 3 shoulder, 2 sternum, and 1 foot) leaving 78 patients with chondrosarcoma of these long bones. All specimens were analysed by experienced dedicated musculoskeletal tumour Pathologists, with the diagnosis and grade given by consensus opinion.

The mean age at presentation was 52 years old (range 10–87 years). The female-to-male ratio was 45 : 33. The most common tumour location was the femur (*n* = 45), followed by the humerus (*n* = 24) and tibia (*n* = 9). Standard radiological investigations included plain radiographs, computed tomography (CT), whole-body bone scintigraphy and magnetic resonance imaging (MRI). All of the tumours were primary chondrosarcomas.

All patients underwent a pre-operative biopsy using a Jamshidi needle, to assist planning of further management. All biopsy approaches were preplanned following discussion with referring surgeons and were performed by specialist radiologists under fluoroscopic or CT guidance. Following departmental policy, all those graded as high grade from the needle biopsy were surgically excised. Tumours graded as low grade were treated either by curettage with bone cement (mostly small, slow-growing, or peri-articular) or by excision (large, rapidly growing or those tumours for which resection and reconstruction was relatively straightforward) dependant upon multidisciplinary discussion. 

The histopathological diagnosis and grade were undertaken in all cases by experienced musculoskeletal pathologists and were based on the light microscopic features according to the World Health Organisation [22]. The histological grade based on needle biopsy and surgical specimens was compared by reviewing the histology reports. Where the biopsy showed a mixed grade, the higher grade was taken as the final grading.

The research was performed following the Declaration of Helsinki principles. Informed consent was obtained from all research subjects.

## 3. Results

After exclusions, 78 long bone chondrosarcoma cases remained. In 67 cases, there was agreement between the needle biopsy histological grade and the final histological grade based on the resection specimen, giving an 86% (67/78) accuracy rate for needle biopsy. In 11 instances, there was a discrepancy in histological grade (Table 1). In 9 of these, the surgical histology revealed a higher grade than that evident from the needle biopsy histology. In 2 cases, a higher grade was attributed to the needle biopsy histology compared to the surgical histology.

When considering the mismatch between clinically low-grade and high-grade lesions, only 5 of the 11 cases with a discrepancy in histological grade were diagnosed as low grade on needle biopsy, but high grade on surgical histology. Therefore, the accuracy of the diagnostic biopsy to distinguish low-grade from high-grade chondrosarcoma was 94% (73/78). 

## 4. Discussion

Chondrosarcoma comprises 10%–15% of all primary bone tumours and can be defined as a malignant tumour whose cells produce hyaline cartilage, usually in a lobular growth pattern. Despite a clear relationship between histological grade and prognosis, the clinical course of chondrosarcoma can be unpredictable [2, 4, 5, 16, 17, 23–25].

Needle biopsy is usually essential for the specific diagnosis of a bone lesion and will often allow an accurate histological grade to be determined, thereby helping guide further management. The literature indicates that the accuracy of histological grading from needle biopsies of bone tumours in general is between 80% and 86% [8–10]. Lerma et al. [26] report 85% concordance between cytology and histology specimens from 39 cases of chondrosarcoma, although Kreicbergs et al. describe the greatest difficulty of all bone tumours in diagnosing chondrosarcoma correctly [9]. In order to achieve this accuracy, precise image guidance is required at the time of biopsy. An experienced pathologist who is able to confirm the diagnosis and provide the tumour grade from small tissue samples is also essential. It should be stressed that biopsy does have associated problems. It is painful, expensive and the biopsy tract requires excision at the time of operation [27, 28]. Therefore when diagnosis and management are not in doubt, biopsy may be avoided.

In our centre, the accuracy of exact histological grading of long bone chondrosarcoma based on image guided needle biopsy was 86% (67/78 cases). However, when classed clinically as either low or high grade, the accuracy was close to 94% (73/78). Grading into these two significant different clinical entities is critical and more important than the exact histological grade, as the treatment regimen for low- and high-grade tumours may markedly differ. Undergrading of tumours may lead to inadequate surgical treatment and increased risk of local recurrence, metastases and subsequent mortality. Overgrading of tumours could bring about inappropriate use of more invasive surgical treatments with the increased risks of surgical complications, psychological morbidity, loss of function, and poor cosmesis. 

On review of the surgical histology, all 78 cases that underwent pre-operative needle biopsy received an appropriate surgical intervention. In all cases the grading of the needle biopsy was critical in determining the treatment plan. The 67 cases where the exact grade of the tumour was correctly identified from the needle biopsy, all received an appropriate surgical intervention. In the 11 cases where there was a mismatch between the needle biopsy and excision specimen histological grading, no inappropriate surgical intervention occurred. The mismatch in only five of these 11 cases meant a reclassification from low grade to high grade. Ten of these tumours were excised primarily and one was treated by curettage with bone cement (this case was ultimately classified as low grade). None of these cases required any subsequent surgical procedures related to their chondrosarcoma. We attribute this successful decision-making to the multidisciplinary approach used. This includes a team of experienced orthopaedic oncology surgeons, radiologists and pathologists, which greatly assists in pre-operative diagnosis and planning. In two of the cases, the histological grade of the needle biopsy overestimated the grade of the tumour. Surgical excision of one of these lesions was undertaken, and the remaining tumour was treated by curettage with bone cement. Both cases are under careful observation with clinical examination and further imaging at regular intervals. For the remaining nine mismatched cases where the needle biopsy suggested a lower-grade diagnosis than the subsequent excision histology revealed, the multidisciplinary team concluded during pre-operative surgical planning that all should subsequently undergo surgical excision rather than curettage with bone cement. In these cases, the treatment decision to err on the side of caution was taken on the basis of the history, clinical examination, and radiographic findings specific to each individual patient as well as the histopathological features. Again, we consider this successful decision making to be a result of discussion between an experienced multidisciplinary team.

We restricted our study to chondrosarcoma of the femur, tibia, and humerus, excluding those tumours occurring in other locations. Such other tumours, particularly those occurring within the pelvis or axial skeleton, may be subject to differing treatment options. Whilst a needle biopsy is still essential in accurately diagnosing the tumour grade, these lesions may be treated differently to their counterparts of identical grade in the long bones that we have included in our study, and direct comparison therefore should be looked upon cautiously.

The most likely explanation for the mismatches in grading that we identified is the well-described heterogeneity of chondrosarcoma lesions and the incumbent sampling difficulties related to this. In the cases where the tumour grade was underestimated, it is likely that the tissue sampled by these needle biopsies represented lower-grade segments of the lesions without sampling the more malignant parts. The reason for the two overestimate discrepancies is unclear, but such cases highlight the potential intra- and interobserver difficulties of chondrosarcoma grade classification. We accept that the mismatches could be explained by human error in interpretation of the specimens at a pathological level, or suboptimal biopsy tissue samples. It is clear that an adequate biopsy tissue sample is essential to make an accurate diagnosis.

We conclude that accurate image-guided biopsy is a very useful adjunct in determining the histological grade of chondrosarcoma and the subsequent treatment plan. At present, a multidisciplinary approach offers the most reliable means of accurately diagnosing and grading of chondrosarcoma of long bones.

## Figures and Tables

**Table 1:** Summary of case histories, histology diagnosis, and management of patients with discrepancy in histological grade (F: femur; H: humerus).

Case	Gender	Age	Bone	Pre-op biopsy	Excision biopsy	Surgery	Local recurrence
1	Male	30	F	Low-grade (II)	High-grade (III)	Excision	No
2	Male	24	F	High-grade (I-II-III)	Low-grade (I-II)	Curettage with bone cement	No
3	Female	77	H	Low-grade (II)	High-grade (I-II-III)	Excision	No
4	Female	54	F	Low-grade (II)	Low-grade (I)	Excision	No
5	Female	10	F	Chondroblastic osteosarcoma	High-grade chondrosarcoma (III)	Excision	No
6	Male	65	F	Low-grade (I)	Low-grade (I-II)	Excision	No
7	Male	51	H	Low-grade (II)	High-grade (II-III)	Excision	No
8	Female	50	F	Low-grade (I)	Low-grade (I-II)	Excision	No
9	Male	28	F	Low-grade (I)	High-grade (II-III)	Excision	No
10	Male	63	F	High-grade (III)	Dedifferentiated chondrosarcoma	Excision	No
11	Female	20	F	Low-grade (I)	Low-grade (I-II)	Excision	No
